# Mycobacterium Chimaera in a Young Patient without Underlying Cardiac Surgery History.

**DOI:** 10.51894/001c.166019

**Published:** 2026-07-31

**Authors:** Kate A. Szczesniak, Rakesh Shah, Malitha Hettiarachchi

**Affiliations:** 1 DMC - Sinai Grace

## 124

### Introduction

Mycobacterium chimaera (M. chimaera), within the Mycobacterium Avium Complex (MAC), is a slow-growing nontuberculous mycobacterium (NTM) species that is most often characterized by nonspecific symptoms of night sweats, fever, and fatigue. Due to the nonspecific symptoms of M. chimaera and the rarity of this infection, delayed or misdiagnosis occurs in these patients. Risk factors of contaminated heater-cooler units used in cardiac surgery have been reported; however, cases without this exposure, such as this case, have been cited less frequently. This case of M. chimaera will present a young patient with dyspnea who was found to have a hemorrhagic pleural effusion.

### Case Description

A 39-year-old female with horseshoe kidney and recent preeclampsia presented with dyspnea and pleuritic, left-sided chest pain radiating to her back. The patient endorsed several days of night sweats, fatigue, chills, and severe headache without leukocytosis. The patient worked in a manufacturing plant with poor sanitary conditions. Imaging showed a large, complex left pleural effusion. Thoracentesis revealed hemorrhagic fluid with a lymphocytic predominance. Differential included malignancy and infection. Additionally, a positive pregnancy test prompted evaluation for ectopic pregnancy and gestational trophoblastic neoplasia. A chest tube was placed, and tPA/dornase was given. The chest tube output became milky and showed triglycerides and cholesterol, suggestive of chylothorax. Infectious workup was negative, except for two sputum cultures that grew M. chimaera. Management was guided by a multidisciplinary team.

### Discussion

The nonspecific presentations of M. chimaera can be difficult to diagnose, especially without risk factors of cardiac surgery. This patient’s M. chimaera diagnosis was complicated by the changing characteristics of the pleural effusion and the positive pregnancy test. Initially, the differential was broad, including autoimmune, malignant, infectious, and gynecologic etiologies. However, the pleural fluid with lymphocytic predominance and the sputum cultures growing M. chimaera narrowed the differential.

### Conclusion

Broad differentials are important for nonspecific presentations, and a high degree of suspicion for rare infections is needed. It is essential to integrate clinical decision-making amongst a multidisciplinary team to identify rare infections. Hopefully, this case helps to make more providers aware of M. chimaera amongst patients without traditionally cited risk factors.

